# The current shapes of cyberviolence in digital relationships

**DOI:** 10.1192/j.eurpsy.2023.146

**Published:** 2023-07-19

**Authors:** I. G. Yilmaz Karaman

**Affiliations:** Psychiatry, Eskişehir Osmangazi University, Faculty of Medicine, Eskişehir, Türkiye

## Abstract

**Abstract:**

Social life has moved toward the digital world in many aspects. As the original inhabitants of virtual life, young people face several adversities while flirting and dating online. The anonymity of the online environment enables aggression without consequences. Cyberviolence has no boundaries as time or place. Thus it may cause psychological distress.

Young people frequently use online dating and social networking sites, which makes them vulnerable to cyber dating violence, sextortion, and revenge porn. Cyberdating violence usually targets young women and girls. It is mainly related to stereotypical gender beliefs: presented as controlling behavior and psychological violence. Survivors of cyber dating violence tend to feel anger, hostility, and loneliness. Sextortion is image-based sexual abuse, which includes threats with intimate pictures or videos. Perpetrators of sextortion can be hackers, ex-partners, or admirers. Exposure to sextortion is related to shame, fear, and helplessness. Former partners may use revenge porn to control one’s behavior. It is another kind of image-based sexual abuse. Perpetrators may use nonconsensual pornography to punish or humiliate their ex-partners.

Cyber victimization is as traumatic as real-life victimization. That results in depression, anxiety, and posttraumatic stress. Moreover, the absence of time and space restrictions can make cyberviolence even more debilitative. I aim to inform European psychiatrists about the current shapes of cyber psychological trauma in the context of online flirting and dating. So that psychiatrists can better understand its content and outcomes, especially while working with young people.

**Disclosure of Interest:**

None Declared

